# Relaxin-Family Peptide Receptors 1 and 2 Are Fully Functional in the Bovine

**DOI:** 10.3389/fphys.2017.00359

**Published:** 2017-06-06

**Authors:** Yanzhenzi Dai, Richard Ivell, Xuan Liu, Dana Janowski, Ravinder Anand-Ivell

**Affiliations:** ^1^School of Biosciences, University of NottinghamNottingham, United Kingdom; ^2^Leibniz Institute for Farm Animal BiologyDummerstorf, Germany; ^3^School of Biological Sciences, University of AdelaideAdelaide, SA, Australia

**Keywords:** relaxin, INSL3, RXFP1, RXFP2, follicle, endometrium, bovine

## Abstract

In most mammals the peptide hormone relaxin is a key physiological component regulating early pregnancy and birth. However, synteny analysis shows that the gene encoding ovarian relaxin-2 is deleted in cows and sheep. While, these ruminants appear to exhibit a relaxin-like physiology, as in other mammals, until now a molecular understanding of this has been lacking. Cloning and expression analysis of the cognate bovine receptor for relaxin, RXFP1, as well as of the structurally related receptor, RXFP2, in female tissues, shows that these are expressed in a similar way to other mammals. RXFP1 transcripts are found in ovarian theca cells, endometrium, and myometrium, whereas RXFP2 transcripts are expressed in ovarian theca cells, oocytes, as well as in myometrium. Transfection of receptor-expressing gene constructs into HEK293 cells indicates that bovine RXFP1 has a greater EC50 at 10–50 nM for porcine or human relaxin, compared to human RXFP1. For bovine RXFP2, in contrast, the EC50 is <1 nM for its cognate ligand, bovine INSL3, but also 10–30 nM for porcine or human relaxin. Functional analysis shows that bovine myometrial cells are able to respond to exogenous relaxin and INSL3 with a significant increase in cAMP. Although expressing mRNA for both RXFP1 and RXFP2, bovine follicular theca cells only respond to INSL3 with a dose-dependent increase in cAMP. Altogether the results suggest that the cow is able to compensate for the missing hormone, and moreover imply that relaxin analogs could offer an important therapeutic option in treating female ruminant infertility.

## Introduction

The relaxin family of peptide hormones includes, besides relaxin (RLN2), also relaxin 3 (RLN3), insulin-like peptide 3 (INSL3), INSL5, and INSL6. Additionally, within higher primates and humans there is RLN1 and INSL4. RLN3 and INSL5 appear in most mammals to be restricted in expression to the brain and gut, respectively, and act through G-protein coupled receptors, referred to as RXFP3 and RXFP4, though the former peptide can also act, but with reduced affinity, at RXFP1 (Bathgate et al., 2006). RLN2 and INSL3 on the other hand are usually expressed in reproductive tissues of both sexes and act at a different subclass of GPCRs, respectively, RXFP1 and RXFP2, which are characterized by large extracellular domains and a superficial similarity to the receptors for the gonadotropin hormones (Bathgate et al., 2006). As their names suggest, the relaxin family of peptides share the basic scaffold of relaxin and insulin, with A and B chains linked by inter- and intra-peptide cysteine sulfhydryl groups, and a connecting C-peptide which may or may not be processed upon *in vivo* synthesis (Ivell et al., 2011).

**Figure 1:**
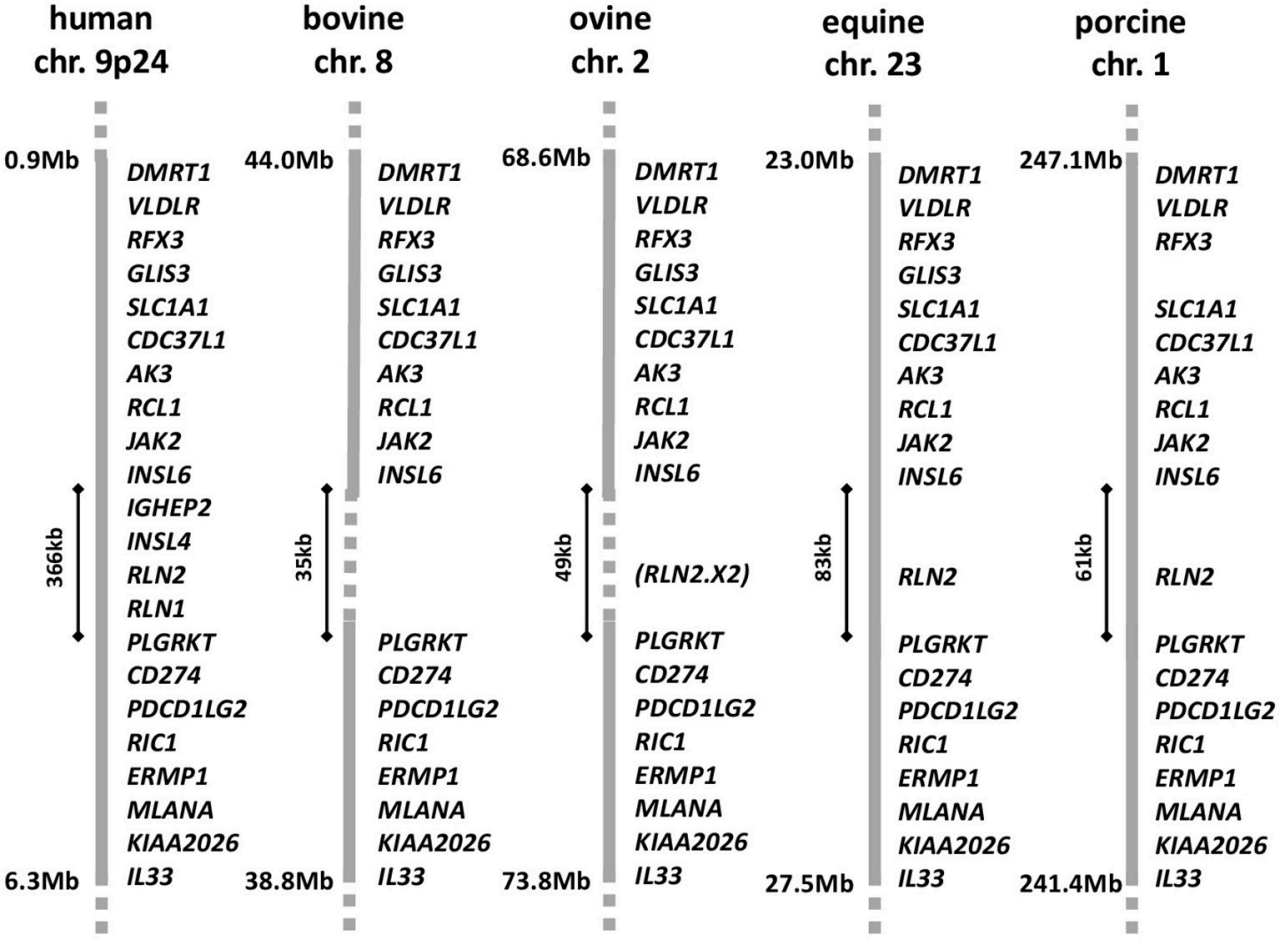
Gene synteny for the corresponding regions of the *DMRT1* locus in the relevant chromosomes for human, bovine, ovine, equine, and porcine genomes, based on the latest annotation lists and sequence information in the NCBI database (release Dec 2016). The region between *INSL6* and *PLGRKT* is expanded in the human genome to accommodate the recent local anthropoid gene duplications giving rise to *INSL4* and *RLN1*. The equivalent region in horses and pigs is shorter and accommodates only a single member of the relaxin family, *RLN2*, representing the primitive mammalian constellation. In sheep and cows this region is reduced again, with a partial or complete deletion of the *RLN2* gene, respectively. In sheep, only a remnant corresponding to the A-chain encoding exon 2 of the *RLN2* gene is present.

For many years there has been a controversy surrounding the existence or not of relaxin in ruminants, including cattle, sheep, and goats (Hartung et al., 1995). A RLN2 homologous molecule is definitely expressed at the mRNA and/or protein levels in related ungulates, such as pigs, camels, llamas, and horses (James et al., 1977; Stewart et al., 1991; Bravo et al., 1996; Hombach-Klonisch et al., 2000; ncbi database: www-ncbi-nlm-nih-gov). But extensive searching by a range of molecular techniques has failed to identify related transcripts in ruminants, except for a partially deleted sequence representing part of the A-chain in sheep (Roche et al., 1993). Proving the absence of a gene even with the extensive genomic sequence available has been difficult, though there appears to be consensus now based on studies of chromosomal synteny that there has been a significant deletion in the bovine genome on chromosome 8, corresponding to where the RLN2 gene should have been located (Figure 1). Why there has been controversy is due to earlier reports at the protein level of relaxin-like immunoreactive entities in bovine tissues and seminal plasma (Fields et al., 1980; Kohsaka et al., 2003), correlating to evident relaxin-like physiologies similar to those described in species like the pig. It would appear that in ruminants some antibodies which had been raised against porcine relaxin may have been cross-reacting with some other possibly related peptide (Ivell et al., 2011). At the time of these earlier studies the existence of other relaxin-like peptides was unknown, and thus could not be controlled for. In spite of the uncertainty relating to the existence or not of a RLN2 homolog in ruminants, there is nevertheless good evidence for a functional relaxin-dependent physiology, at least in cows. Porcine relaxin applied either locally or systemically to beef cows at term elicited a classic softening and dilatation response, thereby influencing calving period (Musah et al., 1987). Moreover, porcine relaxin applied to bovine luteal cells in culture significantly suppressed oxytocin production by these cells (Musah et al., 1990). More recently, relaxin was shown significantly to affect bovine sperm functional parameters (Miah et al., 2011).

It therefore appears that in ruminants such as the cow, there are physiologies which in non-ruminant species are governed by the peptide hormone relaxin, such as parturition, but which appear to proceed relatively normally in the absence of the hormone. Indeed, as a corollary to this, exogenous relaxin or its analogs may offer interesting therapeutic opportunities in ruminants. The present study was undertaken to examine the receptors for relaxin and the related peptide INSL3 in the cow, namely bRXFP1 and bRXFP2, in order to address the question whether in the absence of endogenous ligand bRXFP1 has in any way lost functionality, and/or whether another relaxin-like peptide might be substituting for relaxin in this species.

## Materials and methods

### Bovine tissues and cells

Bovine tissues were obtained from the local slaughterhouse. Tissue collection was in accordance with German, British and European laws and provisions for ethical regulation. Cumulus-oocyte complexes (COCs) were collected and categorized exactly as in Janowski et al. (2012). For oocyte RNA preparation, COCs were mechanically denuded using a glass pipette followed by a 2 min trypsin incubation (1x Trypsin-EDTA solution; T4174; Sigma, Deisenhofen, Germany) to remove residual cumulus cells, then frozen at −80°C. Bovine myometrial cells were prepared from excised uteri exactly as in Heng et al. (2008). The isolated myometrial cells were plated at 6,000 cells/well in a 48-well plate and cultured at 37°C with 5% CO_2_ for 5 days before stimulation. Bovine ovaries without an obvious corpus luteum were selected for the isolation of granulosa and theca interna cells from mid-phase antral follicles (ca. 6–10 mm diameter) using standard procedures (Holtorf et al., 1989; Bathgate et al., 1999). Follicular fluid, including COCs and granulosa cells, was aspirated from the follicles, and collected in granulosa culture medium (DMEM/F-12 (1:1) with 1% bovine fetal serum, 1% antibiotic-antimycotic (ABAM), 2 mM L-glutamine). After manually removing the COCs, the granulosa cells were harvested by centrifugation (150 g, 10 min) and rinsed once with culture medium as above. Four to six millimeters follicles were obtained by dissection. The theca interna layer of mid-phase antral follicles was peeled off and digested with collagenase IV (1 mg/ml) in Medium 199, including trypsin inhibitor (100 μg/ml) at 38°C for 1 h. The isolated theca cells were collected and washed in Medium 199 with 1% ABAM. After an isotonic shock to eliminate red blood cells, the theca cells were seeded at 20,000 cells/well in a 48-well plate in theca culture medium (McCoy's 5A medium, including 1% ABAM, 2 mM L-glutamine, 10 mM HEPES, 0.1% BSA, 10 ng/ml bovine insulin, 5 μg/ml bovine transferrin, and 5 ng/ml sodium selenite) and incubated at 38°C with 5% CO_2_ for ~40 h before stimulation, as indicated. All chemicals and media were from Sigma. Primary bovine mammary epithelial cells (MECs) were prepared according to Yang et al. (2006) and were a generous gift from Professor Hans-Martin Seyfert (Dummerstorf).

### Follicular fluid analysis

In a separate approach, follicular fluids from medium-sized (diameter 3–6 mm) and large (diameter 6–10 mm) antral follicles of freshly collected slaughterhouse ovaries were individually aspirated, flushed with an additional 100 μl of Dulbecco's phosphate buffered saline (DPBS; BioWhittaker, Lonza, Switzerland), and collected into separate tubes. After removal of COCs and brief centrifugation to separate other cells, the supernatants were analyzed for progesterone and estradiol using established in-house radioimmunoassays (Christenson et al., 2013) and for bovine INSL3 by highly specific time-resolved fluorescent immunoassay (TRFIA; Anand-Ivell et al., 2011). Because of inaccuracies in measuring individual antral fluid volumes, hormones were first calculated and compared as total amount per follicle, or as estradiol/progesterone ratio. These individual values were then converted to mean concentrations per follicle by applying average volumes for 3–6 mm follicles of 35 μl and for 6–10 mm follicles of 180 μl after accounting for somatic cells and COCs.

### Oocyte analysis and *In vitro* embryo production

COCs were collected from 3 to 6 mm medium-sized bovine follicles, as above, and subjected to brilliant cresyl blue (BCB) staining to indicate developmental competence, as previously described (Janowski et al., 2012). For *in vitro* embryo production, COCs were pooled, washed in DPBS, and then subjected to *in vitro* maturation exactly as described previously (Janowski et al., 2012). The COCs were then fertilized *in vitro* using frozen-thawed bovine semen, presumptive zygotes manually denuded, and resulting embryos cultured at 38.5°C in 5%CO_2_, 5% O_2_, and 90% N_2_ up to blastocyst stage (day 8; Janowski et al., 2012).

### RT-PCR analysis for RXFP1 and RXFP2 transcripts

Total RNA from endometrial biopsies, myometrial, granulosa, and theca cells, as well as oocytes and embryos was prepared using the RNAqueous-micro total RNA isolation kit (Ambion, Thermo-Fischer, Darmstadt, Germany) according to the manufacturer's instructions, and then single strand cDNA was prepared following the procedure described in Heng et al. (2008). Semi-quantitative RT-PCR was carried out using the primers listed in Supplementary Table 1, designed using the Primer3 software applied to sequences XM_002694415 (bRXFP1) and NM_001304277 (bRXFP2). 0.25 μM of each primer pair was used to amplify 1 μl of cDNA using EmeraldAMP MAX HS PCR master mix (50% of the reaction volume) (Takara-Clontech, Heidelberg, Germany). For each reaction, DNA was denatured at 95°C for 45 s, annealing was carried out at the temperature indicated in the figure legends, with elongation at 72°C for 1 min, for a total of 35 cycles. As control for the integrity of the RNA, all samples were also validated for the expression of the housekeeping gene bovine rp27a (not shown).

All PCR products were analyzed by gel electrophoresis on a 2% agarose gel including 1% ethidium bromide, with visualization using a GelDoc XR system (Bio-Rad, Gladesville, Australia). For PCR product sequencing, the DNA in each gel band was extracted using the MinElute Gel Extraction Kit (Qiagen, Hilden, Germany), and sequenced (Eurofins-MWG, Ebersberg, Germany).

### Bovine RXFP1 and RXFP2 gene constructs and transfection

Preliminary studies, prior to the publication of the bovine genome project, had employed heterologous RT-PCR primers, derived from human sequence information, together with RNA from selected bovine tissues. Initial sequence information obtained was subsequently fully confirmed by the bovine genome project, which is used here for reference. Having confirmed by RT-PCR the correct sequences for the full-length functional bRXFP1 and bRXFP2 transcripts, expression constructs were generated, whereby the signal peptides of the two receptor sequences were replaced by the signal peptide-encoding sequence from the bovine prolactin gene, together with an intermediate short FLAG sequence, exactly as used previously for the expression of the human RXFP1 and RXFP2, and other G-protein coupled receptors (Hsu et al., 2000, 2002). The two final bovine constructs were generated from assembled multiple oligonucleotides (custom gene synthesis by GeneArt, Regensburg, Germany), their sequences confirmed, and inserted into the vector pcDNA3.1/Zeo(+) (Invitrogen) for expression.

For transfection, the Neon electroporation system (Invitrogen) was used: the bRXFP1 or bRXFP2 expression plasmids were mixed with HEK 293T cells in a ratio of ~0.2 μg DNA/50,000 cells/well (48-well plate), and pulsed at 1,300 V for 30 ms to transfect the DNA into the cells. Plasmids were allowed to express for 24 h with the transfected cells cultured in DMEM/F12 (1:1) at 37°C with 5% CO_2_. In parallel, HEK 293T cells which had been permanently transfected with human RXFP1 or human RXFP2 (Heng et al., 2008) were plated at 50,000 cells/well in a 48-well plate, and cultured overnight in DMEM/F12 (1:1) with 10% bovine fetal serum, 2 mM L-glutamine and 1% Penicillin-Streptomycin at 37°C with 5% CO_2_.

### Cell culture and analysis

For the transient and permanently transfected HEK 293T cells and the primary myometrial (6,000 cells/well) and theca cells (20,000 cells/well), after the initial pre-culture period (as above) medium was changed and supplemented or not with 1 mM IBMX (3-isobutyl-1-methylxanthine; Sigma) as indicated (30 min for myometrial cells, 10 min for theca cells). Following this pre-incubation, cells were supplemented or not with various peptides, also as indicated in the figure legends, and incubation continued for a further 20 min. Total cAMP was extracted from the combined cells and culture medium from each well and measured by time-resolved fluorescent immunoassay (TRFIA) as described in Anand-Ivell et al. (2007). cAMP production by the transfected HEK 293 cells was measured directly (range1–243 pmol/ml); cAMP from primary myometrial and theca cells made use of the more sensitive TRFIA employing sample acetylation (detection range 0.04–9.72 pmol/ml).

### Statistics

All data were analyzed and plotted using GraphPad Prism 6. The EC50 values were calculated by non-linear regression of log (agonist) against response (three parameter). All column data were analyzed by ANOVA followed by Neuman-Keuls *post-hoc* test.

## Results

### Primary structure of the bovine RXFP1 and RXFP2 receptors

**Figure 2:**
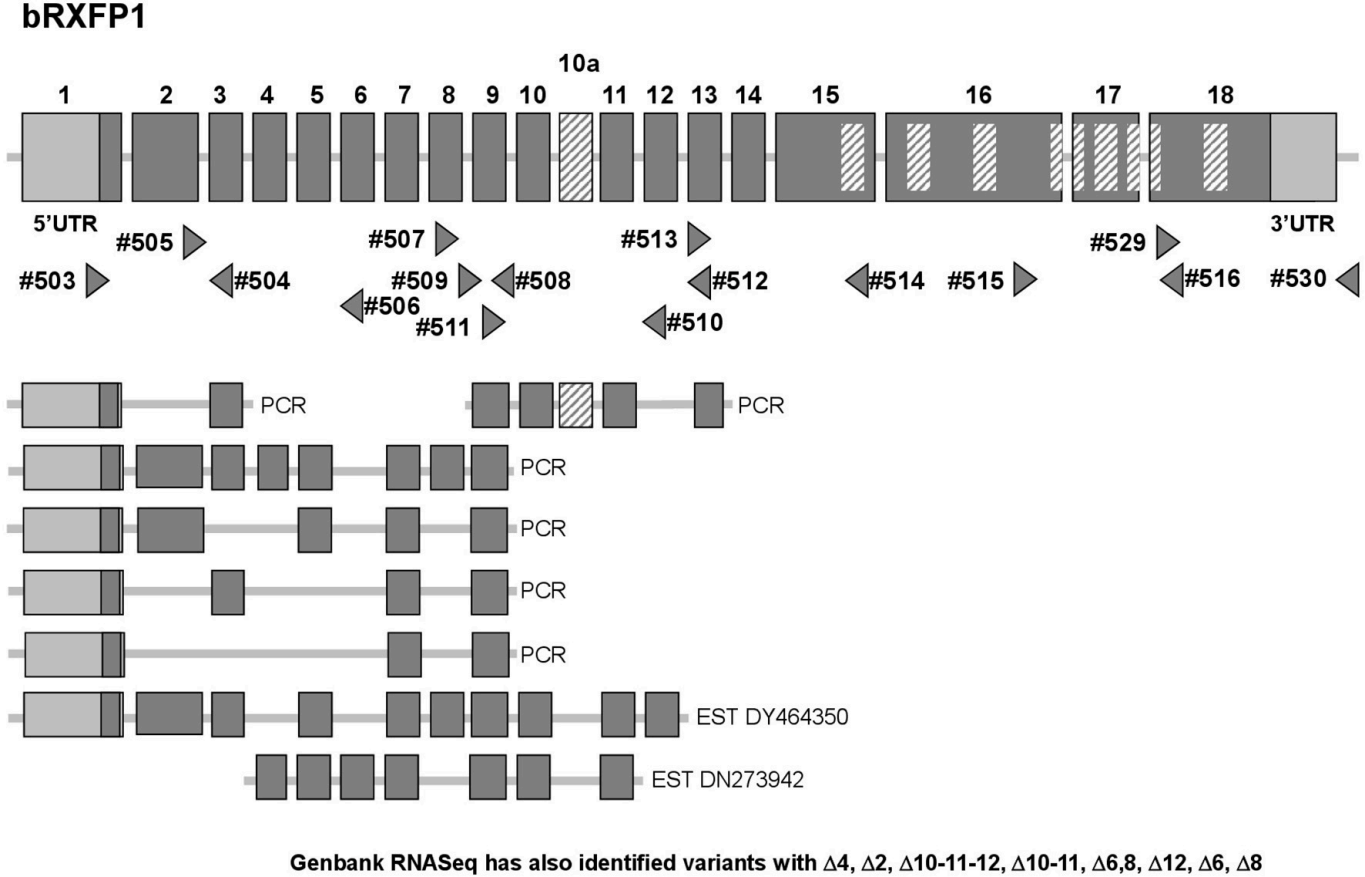
Schematic exon organization of the bovine RXFP1 gene (above) indicating both the position of the novel exon 10a and the locations of the oligonucleotide primers (triangles) used for RT-PCR analysis. The locations of encoded transmembrane regions are indicated by the internal cross-hatched rectangles. The corresponding exon structures of various splice variants identified in this study by RT-PCR, or as pre-existing EST sequences, are indicated below. Additionally, RNASeq analysis (NCBI database; release Dec. 2016) has identified splice variants with the following deletions: Δ4, Δ2, Δ10-11-12, Δ10-11, Δ6, 8, Δ12, Δ6, Δ8.

**Figure 3:**
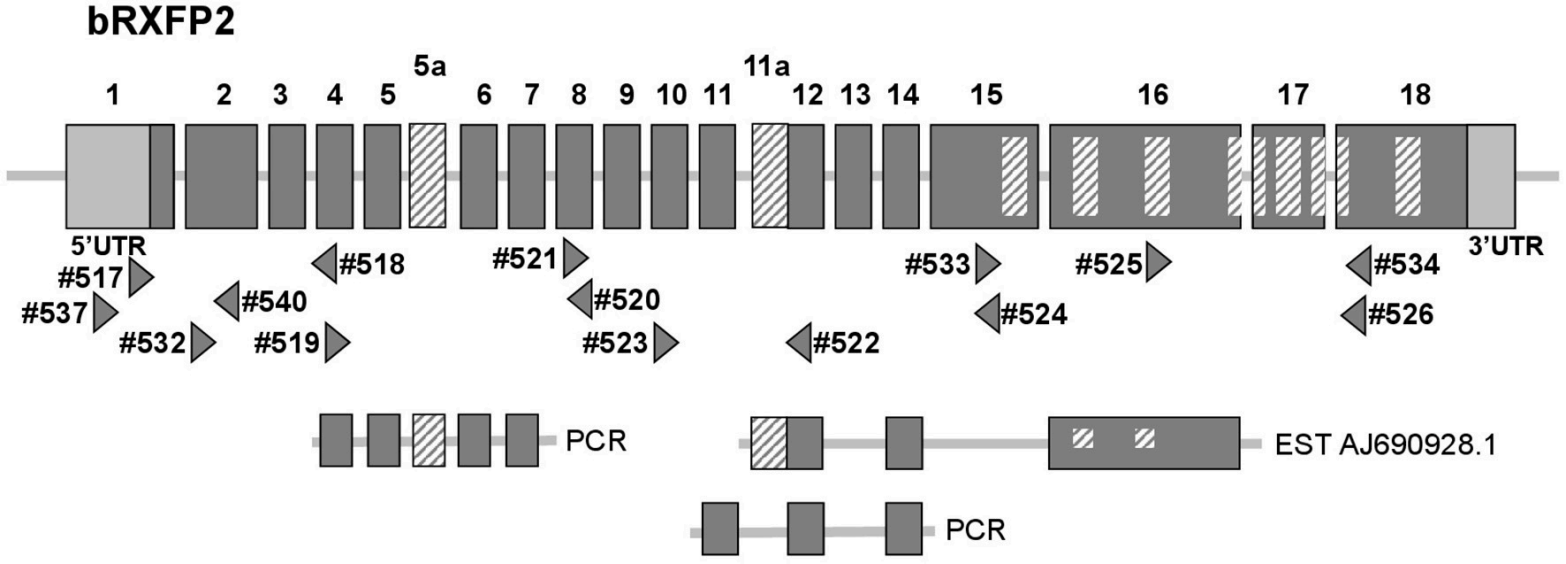
Schematic exon organization of the bovine RXFP2 gene (above) indicating both the position of the novel exons 5a and 11a, and the locations of the oligonucleotide primers (triangles) used for RT-PCR analysis. The locations of encoded transmembrane regions are indicated by the internal cross-hatched rectangles. The corresponding exon structures of various splice variants identified in this study by RT-PCR, or as a pre-existing EST sequence, are indicated below.

At the outset of this project bRXFP1 and bRXFP2 sequence information for the bovine in the NCBI genome database had been missing, incomplete, and in part incorrect, based as it was on partial homologies to other species and application of exon-finding algorithms. RT-PCR strategies were therefore applied using oligonucleotide primers which appeared to be homologous between different species, and using various bovine tissues as a source of potential receptor transcripts. The latest release of the NCBI database (Jan. 2017) now includes complete putative transcript sequences (excluding 5′UTRs) for bRXFP1 (XM_610789.7 and XM_002694415.3) and bRXFP2 (NM_001304277.1), based, however, still on predictions using exon-finding algorithms, homologies to known RXFP1 and RXFP2 sequences in other species, and validated by global RNA-Seq sequence information. Our independent RT-PCR analyses together with genomic BLAST confirmed these two sequences at the expressed transcript level and their corresponding exon-intron structures (Figures 2, 3).

Additionally, our experience from working with RXFP1 and RXFP2 in other species has highlighted the *in vivo* generation of alternatively spliced transcripts for both genes, particularly involving the short 5′ exons representing individual leucine-rich repeats (Anand-Ivell et al., 2006; Heng et al., 2008). The RT-PCR matrix developed to assess the bovine transcripts was designed to identify possible splice variants in expressing cells and tissues (Figures 2, 3). Indeed, in the bovine both genes exhibited multiple splice variants, in which one or more exons are missing, as well as alternative novel exons (Figures 2, 3). It is to be noted that none of the deleted exons, led to a frame-shift or in-frame stop codons. In contrast, where there appears to be the introduction of a novel exon (bRXFP1 exon 10a; bRXFP2 exons 5a and 11a) this indeed leads to an in-frame stop codon in each case. Only where multiple PCR products for a given cell type or tissue consistently indicate the expression of a full-length, putatively functional transcript can we declare that bRXFP1 or bRXFP2 may be functionally expressed in that tissue. Although the RNA-Seq data included in the NCBI database as validation of a particular sequence also suggests the alternative loss of similar exons, the short-sequence nature of RNA-Seq is not sufficient to absolutely discriminate entire transcript structures as implied by the listed so-called RXFP1 “splice variants” (accession numbers XM_010813810-12, XM_010823315-17, XM_005217632, XM_005217635-36, XM_005194978-79, XM_005194981-82).

### Expression of RXFP1, RXFP2, and RLN 3 in cells and tissues of the female bovine reproductive system

**Figure 4:**
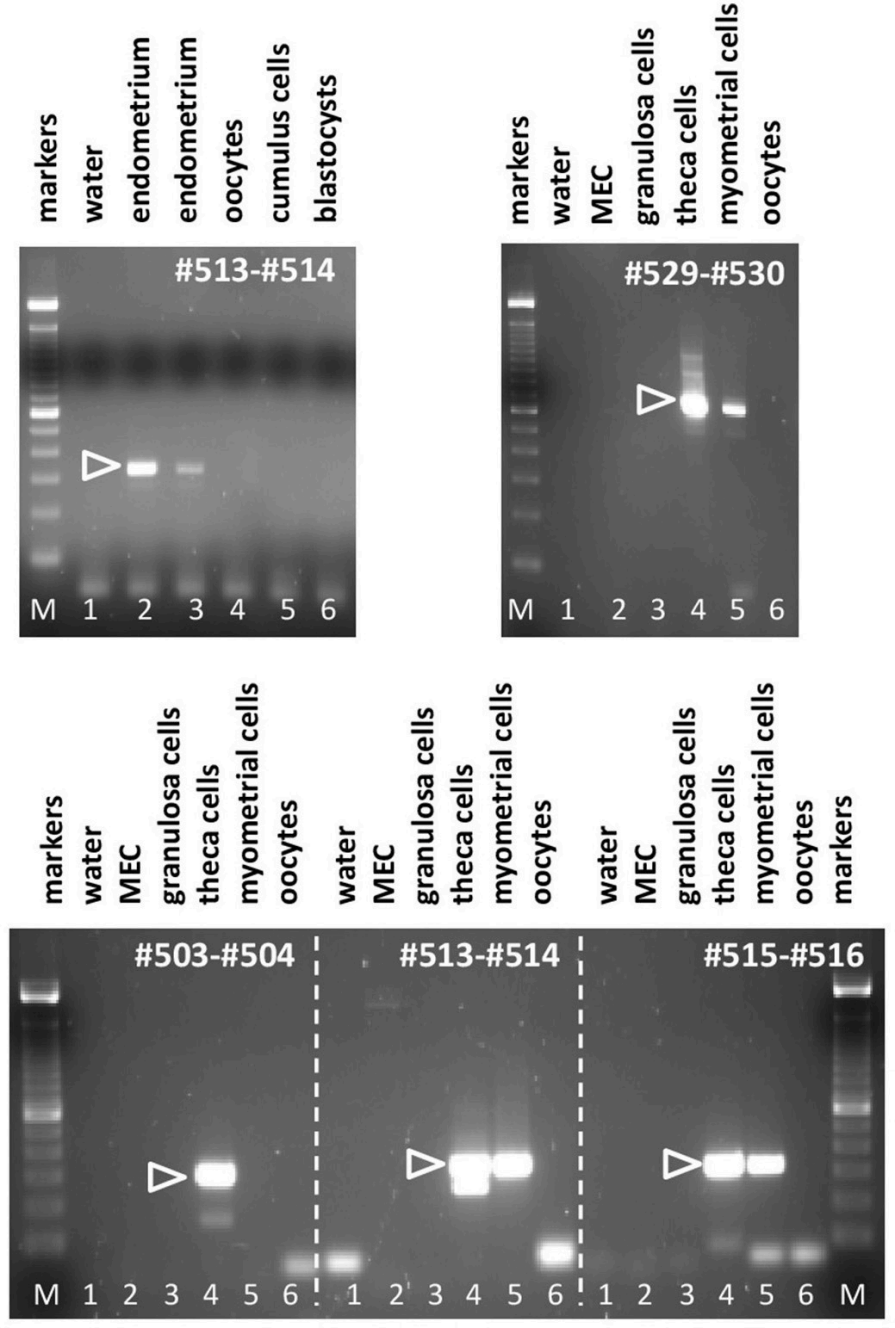
Semi-quantitative RT-PCR analysis for bRXFP1 transcripts of RNA derived from various bovine cells and tissues as indicated, or water controls (lane 1, in all panels). MEC, bovine mammary epithelial cells. PCR primers used are shown in white within each panel. White triangles indicate the position of the correctly spliced PCR product. M, 100 bp DNA ladder used as size markers. PCR conditions (primers, annealing temperature, expected size):#503–#504, 62°C, 301 bp; #513–#514, 62°C, 337 bp; #515–#516, 62°C, 316 bp; #529–#530, 62°C, 692 bp.

**Figure 5:**
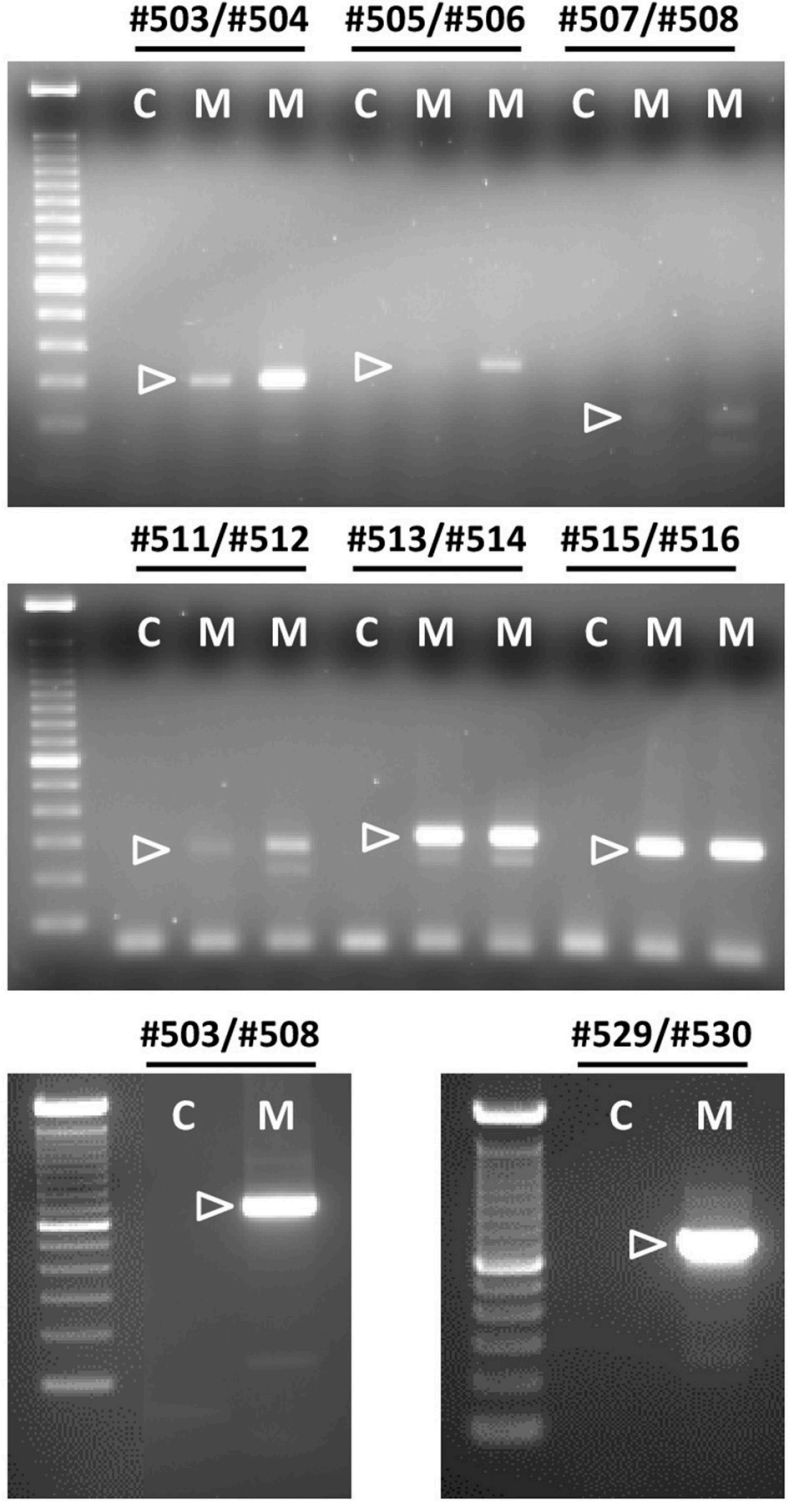
Semi-quantitative RT-PCR analysis for bRXFP1 transcripts of RNA derived from two independent batches of purified bovine myometrial cells (M) as indicated. C, water controls. PCR primers used are shown above each panel. White triangles indicate the position of the correctly spliced PCR product. A 100 bp DNA ladder was used as size markers on the right side of each gel image. PCR conditions (primers, annealing temperature, expected size) as in Figure 4 plus:#505–#506, 62°C, 349 bp; #507–#508, 62°C, 175 bp; #503–#508, 62°C, 753 bp; #511–#512, 62°C, 304 bp.

**Figure 6:**
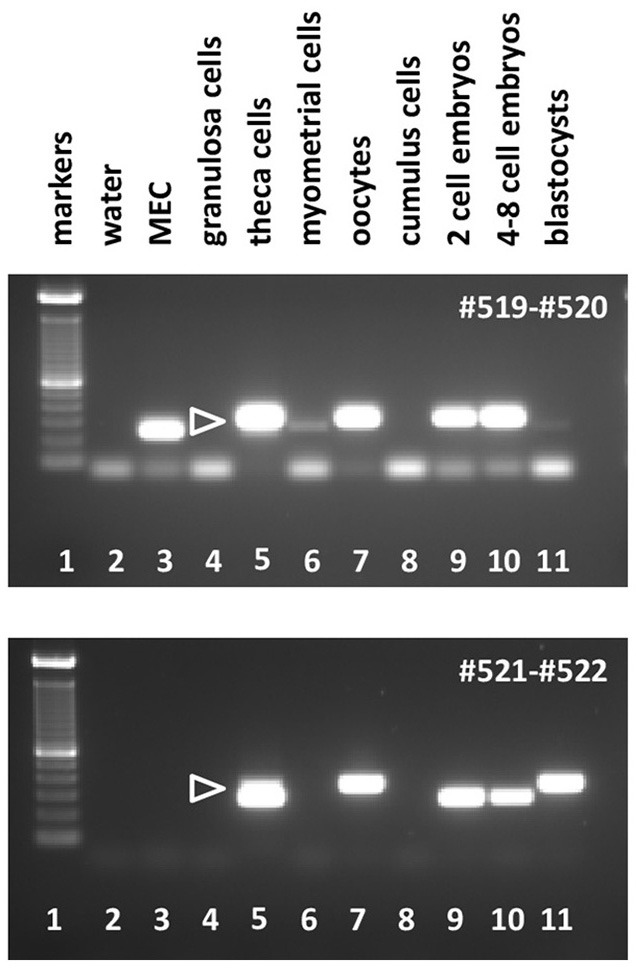
Semi-quantitative RT-PCR analysis for bRXFP2 transcripts of RNA derived from various bovine cells and tissues as indicated, or water controls (lane 2). MEC, bovine mammary epithelial cells. PCR primers used are shown in white within each panel. White triangles indicate the position of the correctly spliced PCR product. A 100 bp DNA ladder is used as size markers in lane 1 within each panel. PCR conditions (primers, annealing temperature, expected size):#519–#520, 62–58°C touchdown, 291 bp; #521–#522, 62–60°C touchdown, 312 bp.

**Figure 7:**
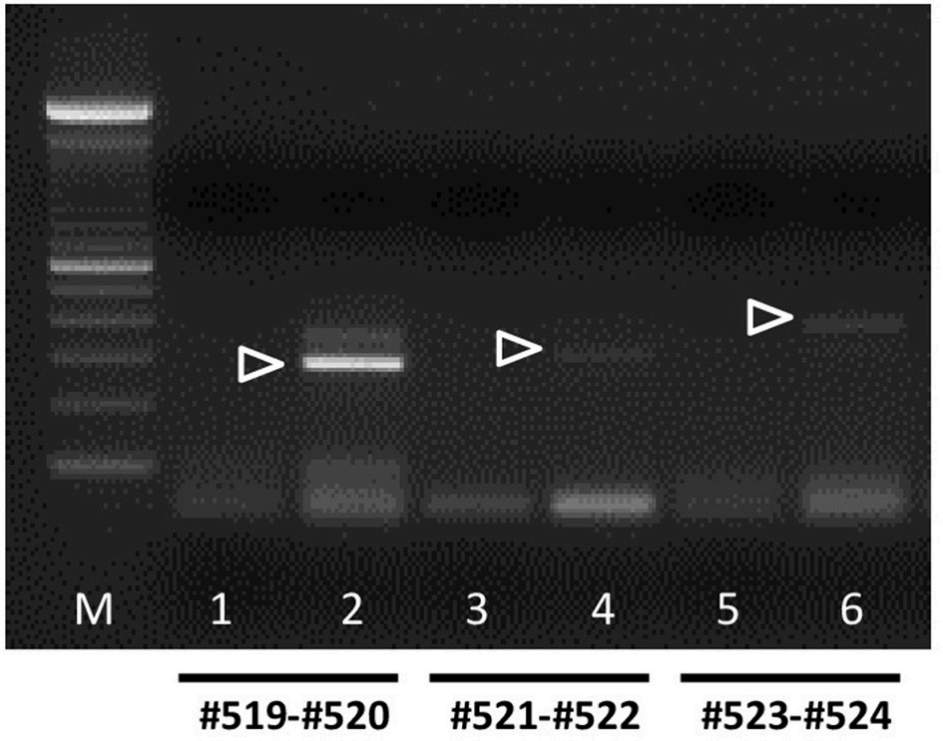
Semi-quantitative RT-PCR analysis for bRXFP2 transcripts of RNA derived from purified bovine oocytes (lanes 2, 4, and 6) compared with water controls (lanes 1, 3, and 5). PCR primers used are shown below the panel. White triangles indicate the position of the correctly spliced PCR product. A 100 bp DNA ladder (M) was used as size markers on the right side of the gel image. PCR conditions (primers, annealing temperature, expected size) as in Figure 6 plus:#523–#524, 62°C, 392 bp.

Applying the matrix of PCR primers indicated in Figures 2, 3, various cells and tissues of the female reproductive system were interrogated for the expression of bRXFP1 (Figures 4, 5) and bRXFP2 (Figures 6, 7). Not all successful PCR primer combinations are illustrated. bRXFP1 is consistently shown to be expressed in the endometrium, myometrial cells, and theca cells, though not in granulosa cells, nor in oocytes and early embryos (not shown). Arrowheads indicate the correctly sized and sequenced PCR products; alternatively sized products, evident in most samples, and mostly as quantitatively minor bands, proved on sequencing to represent splice variants. Myometrial cells are shown in detail in Figure 5, whereby the dominant PCR product always appears to represents the full-length bRXFP1 transcript.

In contrast, bRXFP2 is expressed as correctly sized PCR products (arrowheads) only in theca cells and oocytes, and weakly in myometrial cells. Other sized products, for example, in early embryos, proved upon sequencing to be exclusively splice variants. Detailed RT-PCR analysis for bovine oocytes is shown in Figure 7, where the major product represents the correct full-length transcript, and minor bands represent splice variants.

RT-PCR analysis was additionally undertaken in all cell and tissue samples from the female reproductive system for transcripts representing bovine RLN3, since potentially this could be a local ligand for RXFP1 in these tissues. For no sample could any specific RLN3-related transcripts be identified (not shown).

### INSL3 concentration in bovine follicular fluid

INSL3 concentration was measured in follicular fluids from either medium-sized (diameter 3–6 mm) or large (diameter 6–10 mm) antral follicles from freshly collected ovaries. Total INSL3 per follicle was very variable at 13.2 ± 13.8 ng (*n* = 115) and 24.5 ± 43.1 ng (*n* = 17), respectively (not significantly different). Assuming approximate antral fluid volumes (also taking into account approximate cell mass) for each category of 35 and 180 μl, respectively, this implies *in vivo* concentrations of INSL3 of 376 and 136 ng/ml in bovine follicular fluid, which approximately corresponds to the concentration of INSL3 observed in rat testicular interstitial fluid (ca. 400 ng/ml; ref). There was no association between individual INSL3 amount per follicle and either estradiol or progesterone concentration, nor their ratio (data not shown). Nor was there any significant difference in INSL3 content per follicle and the ability of individual oocytes to stain with BCB or not.

### Stimulation of primary cultures of bovine myometrial and theca cells with RLN and INSL3

**Figure 8:**
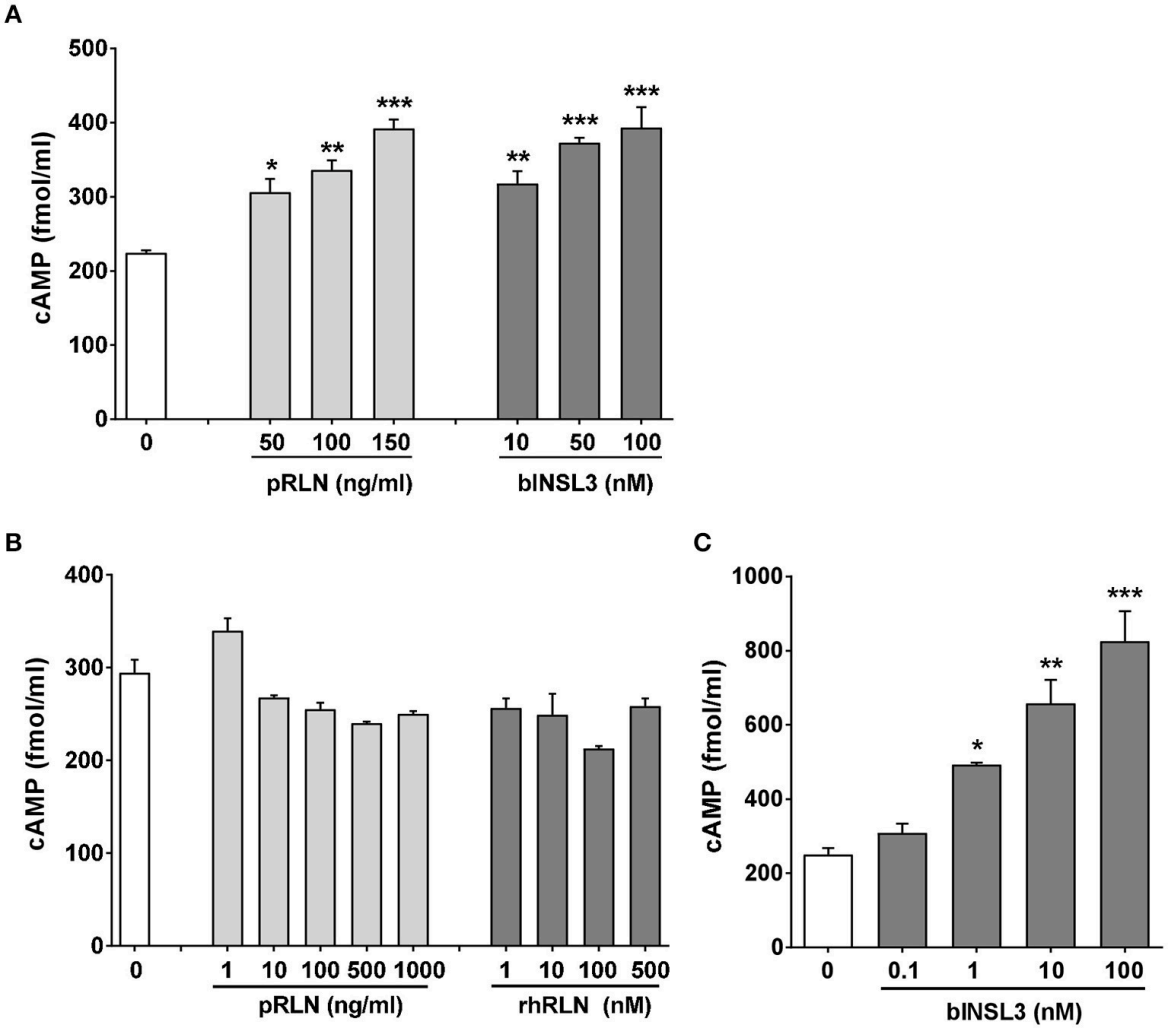
Primary cultures of purified bovine myometrial **(A)** or theca cells **(B,C)** stimulated or not for 20 min with various concentrations of porcine relaxin (pRLN), human recombinant relaxin (rhRLN) or bovine INSL3 in the presence of the phosphodiesterase inhibitor IBMX at 1 mM, measuring cAMP production as endpoint. Significant difference from basal is indicated by asterisks (^*^p < 0.05; ^**^p < 0.01; ^***^p < 0.001).

From the RNA analysis, two cell types appeared to express both bRXFP1 and bRXFP2 as apparently full-length functional transcripts. To confirm this, primary myometrial cell cultures were stimulated with either porcine RLN or bovine INSL3, measuring acute cAMP production in the presence of the phosphodiesterase inhibitor IBMX as endpoint. For both peptides a significant stimulation was observed at all concentrations applied (Figure 8A). For cultured bovine theca cells, INSL3 (Figure 8C), but neither human nor porcine relaxin (Figure 8B), was able significantly to stimulate cAMP production in a dose-dependent manner. The fold-increase in cAMP observed was similar to that seen for human primary cell types (Anand-Ivell et al., 2007; Heng et al., 2008).

### Transfection and stimulation of bovine RXFP1 and RXFP2

**Figure 9:**
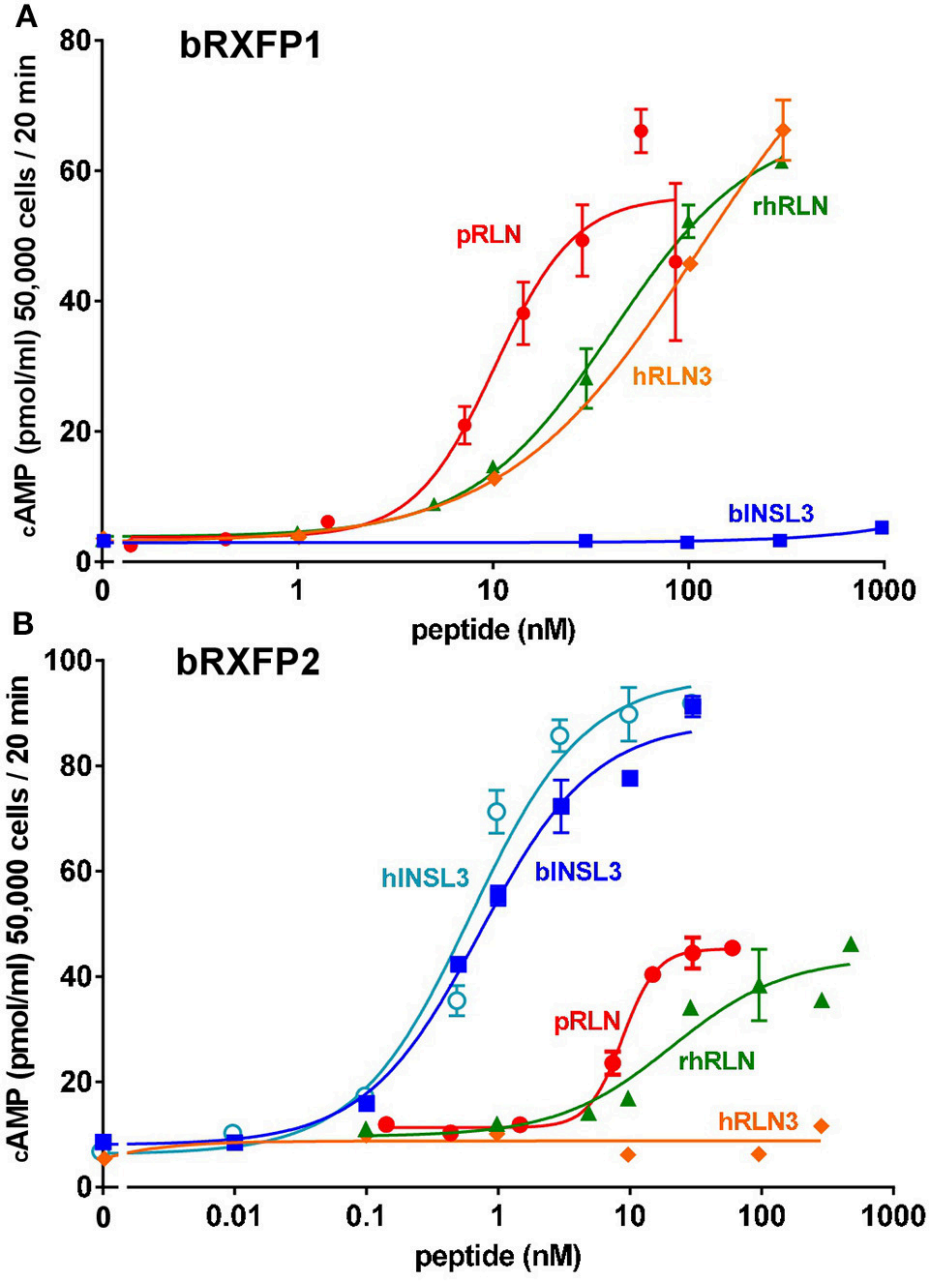
Dose-response curves for cAMP production by HEK293T cells transiently transfected with either bRXFP1 **(A)** or bRXFP2 **(B)** and stimulated by increasing concentrations of porcine relaxin (pRLN), recombinant human relaxin (rhRLN), relaxin 3 (RLN3), bovine INSL3 (bINSL3), or human INSL3 (hINSL3), as indicated.

Full-length transcripts for bRXFP1 and bRXFP2 were synthesized, replacing the natural leader sequences with that of the bovine prolactin gene, as previously described for the human equivalent receptors (Hsu et al., 2002), and subcloned into the expression vector pcDNA3.1/Zeo (+). These plasmids were transiently transfected into HEK293T cells and acutely stimulated with various putative ligands as indicated in Figures 9A,B. For bRXFP1 only porcine RLN, recombinant human RLN2 and human RLN3 showed any stimulation within the range of concentration applied (Figure 9A). For bRXFP2 only INSL3 was effective at low concentrations, though porcine RLN, rhRLN, but not RLN3, could stimulate at very high concentration (Figure 9B). The amounts of cAMP generated were similar to previously published studies for the transfected human receptors (e.g., Hsu et al., 2002; Heng et al., 2008). In parallel, similar stimulations were carried out using permanently transfected cells expressing human RXFP1 (hRXFP1) and hRXFP2 (Table 1). Whereas, hRXFP1 indicated EC50s for porcine and human RLN of <1 nM, the corresponding values for bRXFP1 were 10.8 and 48.7 nM, respectively. Similarly for RLN3, the EC50 value differed by almost one order of magnitude (Table 1). For RXFP2, in contrast, EC50 values were largely comparable between human and bovine receptors (Table 1). For its cognate ligand INSL3, whether human or bovine, the EC50s were consistently 0.6–0.7 nM, and for RLN of different sources EC50 values ranged from 10 to 50 nM. hRLN3 was unable to stimulate either hRXFP2 or bRXFP2 (Table 1).

**Table 1:** EC50 values for human and bovine RXFP1 and RXFP2.

**Peptide**	**bRXFP1**	**hRXFP1**	**bRXFP2**	**hRXFP2**
pRLN	10.8 nM^a^	0.4 nM^a^	10.5 nM^a^	>12 nM^a^^,^^b^
rhRLN	48.7 nM	0.9 nM	27.3 nM	53.9 nM
hRLN3	97.9 nM	14.6 nM	n.d.^c^	n.d.^c^
bINSL3	>1,000 nM^b^	n.d.^c^	0.7 nM	0.6 nM
hINSL3	n.d.^c^	n.d.^c^	0.6 nM	0.7 nM
insulin	n.d.^c^		n.d.^c^	
IGF1	n.d.^c^		n.d.^c^	
IGF2	n.d.^c^		n.d.^c^	

a *Concentrations for porcine RLN have been converted to nM assuming a MW of 6 kD and a purity of 85%*.

b *dose-response curve had not reached an asymptote at the highest concentration used*.

c *n.d., Not determined; insufficient stimulation at highest concentration applied*.

## Discussion

Possibly because of the relatively high incidence of dystocia in cows (Mee, 2008) and because of the marked relaxin-dependent physiology in pigs as a closely related species, the pursuit of a relaxin physiology in cows has pre-occupied reproductive scientists for several decades (reviewed in Hartung et al., 1995). Whilst it now appears that the RLN ligand is not expressed in ruminants (Figure 1), the discovery of other relaxin-like peptides has reopened the question. To address this, we have cloned and studied the expression in the bovine female reproductive system of the two related receptors bRXFP1 and bRXFP2, which in other species specifically respond to RLN and the structurally closely related INSL3. As anticipated from other recent studies on the role of INSL3 in the bovine ovary (Glister et al., 2013), bRXFP2 conforms structurally and functionally to RXFP2 from other species, showing high specificity toward INSL3 (EC50 < 1 nM), and modest cross-reactivity with either porcine or human RLN (EC50, 10–30 nM). It is expressed at a high level in ovarian theca cells consistent with its role to promote androstenedione synthesis in these cells (Glister et al., 2013; Anand-Ivell and Ivell, 2014). It is also expressed in oocytes, where it may be involved in oocyte physiology (Kawamura et al., 2004), and at a low level in bovine myometrial cells, as shown previously also for the human (Heng et al., 2008). In the absence of any circulating relaxin in the cow, bRXFP2 is exclusively activated only by INSL3. In transfected cell systems bRXFP2 signals through adenylyl cyclase, as does hRXFP2 (Heng et al., 2008), leading to an increase in intracellular cAMP. Assessment of primary myometrial and theca cell cultures also suggests an activation of adenylyl cyclase, although this does not exclude activation of other protein kinase A-dependent and -independent signaling pathways (Halls et al., 2015).

The observation of significant amounts of bRXFP2 transcripts in bovine oocytes is in agreement with observations in rodents (Kawamura et al., 2004; Xue et al., 2014), as well as with corresponding findings from the male gametes of rodents and humans (Anand-Ivell et al., 2006; Filonzi et al., 2007). However, the high apparent concentration of INSL3 in bovine follicular fluid, together with the measured EC50 for bRXFP2, would suggest that the oocyte receptor is either permanently occupied through the antral phase, or permanently desensitized, or both. It is possible that oocyte bRXFP2 plays an important role at an earlier time-point in oocyte development before INSL3 is markedly expressed in the follicle. Alternatively, it may become important only after ovulation when the oocyte is liberated from the high INSL3 environment. Further research will be needed to examine these options.

An earlier study also showed significant amounts of INSL3 mRNA within the bovine corpus luteum (Bathgate et al., 1996). Although, we did not revisit this structure in the present study, future research will also focus on the role of both RXFP1 and RXFP2 dependent physiology in luteal function.

The bRXFP1 receptor also appears to be functional, in transfected cell systems responding to both porcine RLN and hRLN, albeit with markedly increased EC50 values (10–50 nM). Significantly, it also responds to hRLN3 (which retains 96% amino acid homology to bRLN3) though again with a markedly increased EC50 (~100 nM) compared to the human receptor (~15 nM). This is important since it has been suggested that at least in the brain RXFP1 may be a natural receptor for RLN3 (Meadows and Byrnes, 2015). It should be noted, however, that higher EC50 values may be less relevant in cell systems where receptor and expressed ligand are closely juxtaposed. Since we could find no evidence for local RLN3 expression in cells of the female reproductive system, it seems unlikely that RLN3 may be a significant ligand for bRXFP1 in the female reproductive system *in vivo*. Nevertheless, bRXFP1 remains functional, as shown here by its apparently specific response in myometrial cells, though not in theca cells, and by past studies showing specific effects on bovine luteal cells in culture (Musah et al., 1990) and in cervical softening and dilatation *in vivo* (Musah et al., 1987).

The question remains, if the specific and unique ligand for a receptor has been lost by mutation during ruminant evolution, why is it that the receptor remains functional, albeit with an increased EC50 for an exogenous ligand, and is still expressed in those cell types known to respond to RLN in other species? In otherwise similar circumstances one would expect rather to see an accumulation of missense mutations and deletions in such a receptor gene or in its control regions, leading to a loss of function and/or expression. This suggests that bRXFP1 is still functioning *in vivo* though with alternative activation mechanisms. Besides, the possible selective effect of a brain relaxin-3/RXFP1 system noted above, one possibility could be that there are other non-relaxin ligands *in vivo*, possibly similar to the C1q-tumor necrosis factor-related protein 8 (CTRP8) peptide recently reported (Glogowska et al., 2013), or like whatever molecules might activate the related orphan LGRs (Hsu et al., 2000). An alternative possibility arises from the known properties of hRXFP1, and probably hRXFP2, to form homo- and hetero-dimers, also with other GPCRs, such as the angiotensin receptor (Chow et al., 2014; Halls et al., 2015), which in the absence of RLN might then be activated, for example, by INSL3 alone. This would be particularly relevant in those cells, such as myometrial or theca cells, where bRXFP1 and bRXFP2 appear to be co-expressed. This might also explain the prevalence of the various splice variants for bRXFP1 and bRXFP2 in which predominantly exons representing individual leucine-rich repeats from the N-terminal extracellular domains of the receptors are missing, but without inducing any frame-shift. Such variants could result in translated proteins with defective ligand-binding and/or signaling properties, but whose transmembrane and intracellular domains are intact. Such transcript variants might act as inhibitors in a receptor dimerization situation (Kern and Bryant-Greenwood, 2009). Further research will be necessary to elucidate these details.

In conclusion, we have shown that cows indeed express functional bRXFP1 receptors in cells of the female reproductive system comparable to those in non-ruminant species. Although ruminants do not express the normal ligand for this receptor, the results strongly imply that cows would be amenable to exogenous administration of RLN or its analogs in a therapeutic context, as was suggested some 30 years ago for the treatment of dystocia (Musah et al., 1987).

## Author contributions

YD carried out most of the experimental work, and assisted in conception, writing and interpretation of the results. RI was responsible for conception of the project, bioinformatic analysis, interpretation of results, and writing of the manuscript. XL and DJ contributed to different experimental aspects of the project, as well as to the interpretation of results. RA had overall responsibility for the project, contributed to conception, experimental design, individual experiments, results interpretation, and writing of the manuscript.

### Conflict of interest statement

YD was supported in part by Immundiagnostik GmbH, Germany, who however had no influence on any aspect of the research. The other authors declare that the research was conducted in the absence of any commercial or financial relationships that could be construed as a potential conflict of interest.
