# Supplementary material for: Relaxin-Family Peptide Receptors 1 and 2 Are Fully Functional in the Bovine
Source: Front Physiol. 2017 Jun 6;8:359. doi: 10.3389/fphys.2017.00359 (PMC5459885; doi:10.3389/fphys.2017.00359)
Supplement: Supplementary file 1 [file Table1.DOCX]

*Supplementary Table 1*

**List of oligonucleotide primers used for RT-PCR reactions.**

| **No.** | **Gene** | **Sequence** |
| --- | --- | --- |
| #503 | *RXFP1* | **tgacatctcgtcctgtcttcc** |
| #504 | *RXFP1* | **ggagaaccgaccaagcattc** |
| #505 | *RXFP1* | **cgaggacaactgtggagacatc** |
| #506 | *RXFP1* | **cagtcctcggaaagcatagaca** |
| #507 | *RXFP1* | **gaagccgggtgtttttgaag** |
| #508 | *RXFP1* | **gggcatgtgctgacaaagag** |
| #509 | *RXFP1* | **taatgtccttacccgcttgc** |
| #510 | *RXFP1* | **ctagtttctggagaggtgcaaatg** |
| #511 | *RXFP1* | **ccgcttgcctgacaaatctc** |
| #512 | *RXFP1* | **ggcttgaattgtctggattgg** |
| #513 | *RXFP1* | **cccaatccagacaattcaagc** |
| #514 | *RXFP1* | **ggcatgcagcttgttctcag** |
| #515 | *RXFP1* | **ggagcccagatttattcagtgg** |
| #516 | *RXFP1* | **gaatcgggttcaaagcactgt** |
| #517 | *RXFP2* | **cttcagccgcagattgattg** |
| #518 | *RXFP2* | **cagttcctttgcacacacagc** |
| #519 | *RXFP2* | **gtgccagtggtttctagcaatg** |
| #520 | *RXFP2* | **caaccgctgtgaaattctgc** |
| #521 | *RXFP2* | **gcagaatttcacagcggttg** |
| #522 | *RXFP2* | **tgaaaatgtggggtggtagc** |
| #523 | *RXFP2* | **tccacctttctgtcgtgcag** |
| #524 | *RXFP2* | **ccgtcagtcaagggcataca** |
| #525 | *RXFP2* | **gttcctggccgttgtctttc** |
| #526 | *RXFP2* | **acgacccaggaggtgattgt** |
| #529 | *RXFP1* | **acagtgctttgaacccgattc** |
| #530 | *RXFP1* | **gtttcaagacaggcaaagctg** |
| #532 | *RXFP2* | **tgtgggaatctctcccagtg** |
| #533 | *RXFP2* | **tgtatgcccttgactgacgg** |
| #534 | *RXFP2* | **acgacccaggaggtgattgt** |
| #537 | *RXFP2* | **tcagttgctgtgaacccattg** |
| #540 | *RXFP2* | **gccgtcacagtgaaaagcac** |
| #527 | *bRLN3* | **gggcaacagcctatggagtg** |
| #528 | *bRLN3* | **gagtctgccgatttcgctct** |
| #501 | *rpS27a* | **tcctcctgaccagcaaagactg** |
| #502 | *rpS27a* | **tgcttgttcttcttgggagtgg** |
